# Liberalising agricultural policy for sugar in Europe risks damaging public health

**DOI:** 10.1136/bmj.h5085

**Published:** 2015-10-27

**Authors:** Emilie K Aguirre, Oliver T Mytton, Pablo Monsivais

**Affiliations:** 1 UKCRC Centre for Diet and Activity Research, School of Clinical Medicine, University of Cambridge, Cambridge CB2 0QQ, UK

## Abstract

**Emilie Aguirre and colleagues** discuss what changes to Europe’s agricultural policy might mean for our health

**Figure:**
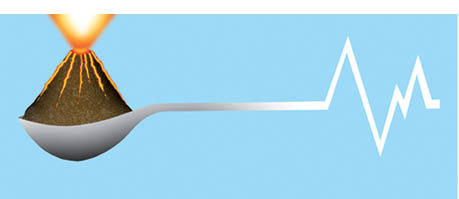


Concerns about the health effects of dietary sugars have recently taken centre stage, reflecting an emerging understanding of the importance of sugars, and particularly sugary drinks, in the development of obesity and diabetes.^1^ ^2^ ^3^ ^4^ Recent research estimates consumption of sugar sweetened beverages will cause about 80 000 excess cases of type 2 diabetes in the UK over 10 years.^1^ In early 2015, the World Health Organization recommended intake of free sugars should be less than 10% of daily calories, and preferably below 5%.^5^ In July, the UK Scientific Advisory Committee on Nutrition halved its recommendation for free sugars to no more than 5% of daily calories.^6^

Dietary sugars (non-milk extrinsic sugars) made up about 15% and 12% of dietary calories among UK children and adults, respectively, in 2012.^7^ Recently there have been calls for action to reduce sugar consumption, including voluntary industry reformulation and taxes or warning labels on sugary foods.^8^ ^9^ ^10^ Earlier this year, Public Health England proposed a series of evidence informed measures to reduce sugar consumption.^11^

So far, relatively little attention has been given to important structural factors, including in agriculture, which influence sugar consumption in the UK.^12^ ^13^ ^14^ However, agricultural policy, through its effect on price and availability of foods, is known to be an important determinant of health.^12^ ^14^ ^15^ ^16^ ^17^ ^18^ ^19^ The European common agricultural policy has historically protected the European sucrose (sugar beet) industry through interventions that have kept commodity prices high and prevented foreign imports. For the past decade, the EU has been phasing out these protections (“liberalisation”). This process will be nearly complete by 2017. We describe the effect liberalisation may have on the production, price, and addition of sugars to processed foods.

**Definitions of terms**

*Sugar—*An umbrella term to describe all monosaccharides and disaccharides. It includes both sucrose and high fructose corn syrup
*Sucrose*—A disaccharide made up of 50% glucose and 50% fructose, typically derived from sugar beet or sugar cane
*High fructose corn syrup—*An alternative to sucrose often used in food manufacturing. It contains a mixture of fructose (between 42% and 60%) and glucose. Although there has been much discussion about the health effects of fructose, the Scientific Advisory Committee on Nutrition recently concluded that there was insufficient evidence to show that they differed from those of other sugars^6^
*Free sugars—*The preferred term for sugars of health concern.^5^ ^6^ It includes any sugars added to foods by the manufacturer, cook, or consumer plus sugars naturally present in honey, syrups, fruit juices, and fruit concentrates.^6^ Free sugar does not include sugars that are within the cellular matrix of fruit and vegetables
*Non-milk extrinsic sugars—*All sugars not contained within the cellular structure of foods other than milk and milk products. Unlike free sugar, it includes fruit sugars found in stewed, dried, or tinned fruit

## How the common agricultural policy has shaped diets

The common agriculture policy was enacted in 1962, when Europe was emerging from food shortages after the second world war. Its primary aims were to increase agricultural productivity, ensure a fair standard of living for farmers, stabilise markets, ensure availability of energy dense food supplies, and establish reasonable consumer prices. Its aims have not evolved as understanding of nutrition for health has improved and as new public health concerns have emerged, including obesity and diabetes. The policy sets common rules in agriculture for all EU member states. These have primarily been concerned with using market interventions to control the supply and price of many agricultural products (including dairy, red meats, sugar, cereals, and vegetables fats). In doing so, the policy has promoted overproduction of these products, shaping European diets in ways that may have been detrimental for public health.^15^

Sucrose has been among the most protected European agricultural products. These protections have benefited sugar beet processors, who have in turn influenced sugar policies.^20^ Protection consisted of a combination of import tariffs, minimum price guarantees, production quotas, and export subsidies. Import tariffs effectively prevented cheaper sucrose being imported from outside Europe. Minimum price guarantees and production quotas ensured European producers were paid substantially above the world price for sucrose produced within the quota. Moreover, export subsidies made it profitable to produce an excess of sucrose (above quotas) and export this to other countries, even when production was costly compared with the world price. Because sucrose was so profitable, the policy soon led to overproduction. The EU also maintained a production cap on high fructose corn syrup of about 5% of all sugar production, affording additional protections to the European sugar beet industry by preventing large scale replacement of sucrose with high fructose corn syrup.

These policies facilitated the growth and profitability of the European sugar industry such that it now includes five of the world’s 10 largest sugar producers.^20^ They also ensured sucrose was the predominant sugar and that its price was kept relatively high compared with the world price.^20^ In 2012, before the reforms, the European price was about €700 (£500; $800) per tonne compared with a world price of about €400 per tonne.^21^

## Liberalising the sugar sector

Initial sugar reforms in 2006 reduced the minimum price guarantee and eliminated export subsidies. One study estimated that reducing the price guarantee could lead to a 7.5% increase in consumption of sugar sweetened drinks in France.^22^ Subsequent reforms, which started in 2013, go much further and will almost fully liberalise the sugar market in Europe, culminating with the elimination of production quotas and minimum price guarantees in 2017. When the reforms were introduced, the European Commission predicted that the commodity (or wholesale) price of sugar would drop substantially, production of high fructose corn syrup would treble, and production of sugars overall would increase by around 15% in the decade after quotas end.^21^ Early indications suggest these predictions are broadly accurate. The price of European sucrose has fallen about 40% to around €400 a tonne, with analysts expecting an increase of around 20% in sugar production after 2017.^23^ ^24^ The main players in the European sugar industry are growing larger and preparing to increase production to remain competitive. For example, in May 2015 Europe’s second largest sugar producer, Tereos, purchased the sugar distribution business of a UK based baked goods company, citing the reform as necessitating this consolidation. Tereos has also stated it will increase sugar production by 20% once quotas are abolished.^24^ Without price controls and quotas the only way for the European sugar industry to remain profitable is by increasing production.

## Unintended effects on sugar consumption 

Sugar supply and consumption in the UK has declined over the past 50 years (fig 1). The effect of the 2006 reforms on consumption is unclear, but the new set of sugar reforms go much further and may be more liable to increase sugar consumption through a variety of mechanisms. For example, lowering the cost of sugars to food processors will make it more economically viable to incorporate sugars into processed foods as an easy, inexpensive means of increasing palatability, potentially resulting in higher sugar content in foods that already contain sugars.

**Figure fig1:**
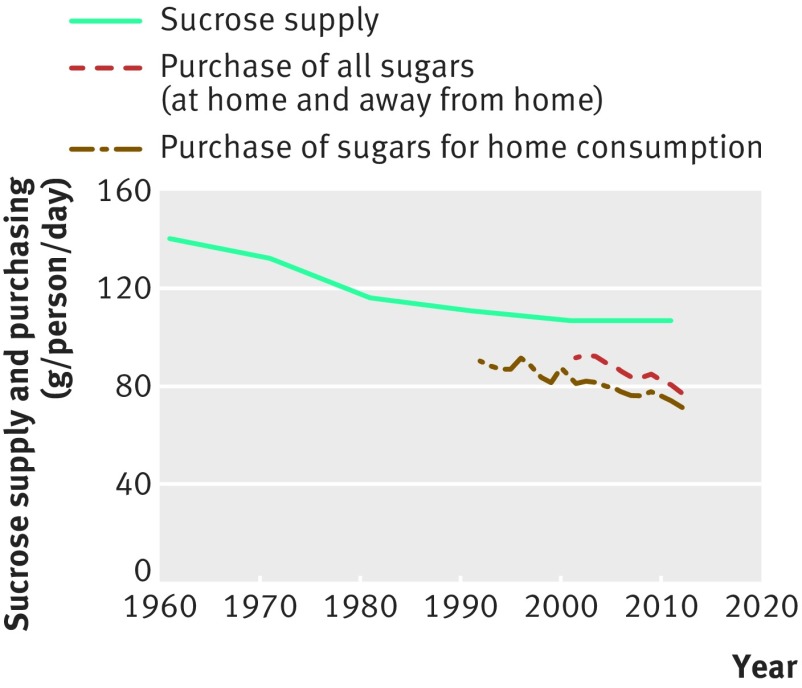
**Fig 1** Sucrose supply and consumption in the UK, 1961-2012. Supply is the total sucrose produced plus imports minus exports, adjusted to provide per capita estimates. Purchase of sugars (non-milk extrinsic sugars) is based on household food purchase, adjusted to provide per capita estimates. From 1990 to 2001, purchase data are from the National Food Survey. Purchase data from 2002 to 2012 are from the Living Cost and Food Survey, formerly known as the Expenditure and Food Survey

The price drop in sugar and increased availability of high fructose corn syrup may also result in sugars being added to a broader range of foods. Apart from sweetness, high fructose corn syrup has benefits for flavour, stability, freshness, texture, pourability, and consistency, and it can be used in both sweet foods and some savoury foods (such as ketchup).^25^ Use of high fructose corn syrup in Europe is relatively low at present but the removal of the production cap in 2017 will make it feasible to produce and use. The United States shows the potential effect of this change. The US government declared high fructose corn syrup to be “generally recognized as safe” in 1983, removing any restraint on its use. Following this, sugary drink manufacturers replaced sucrose with cheaper high fructose corn syrup.^26^ In the 30 years since, there has been a long term decline in the price of carbonated soft drinks relative to food (fig 2). By contrast in the UK, where sucrose remained the predominant sweetener, the price of soft drinks relative to food has risen. Moreover, in the US sugar consumption increased by 20% over the 15 years after the introduction of high fructose corn syrup, even though sucrose consumption declined.^26^ Other differences between the US and Europe make it difficult to predict whether Europe would see a similar size effect.

**Figure fig2:**
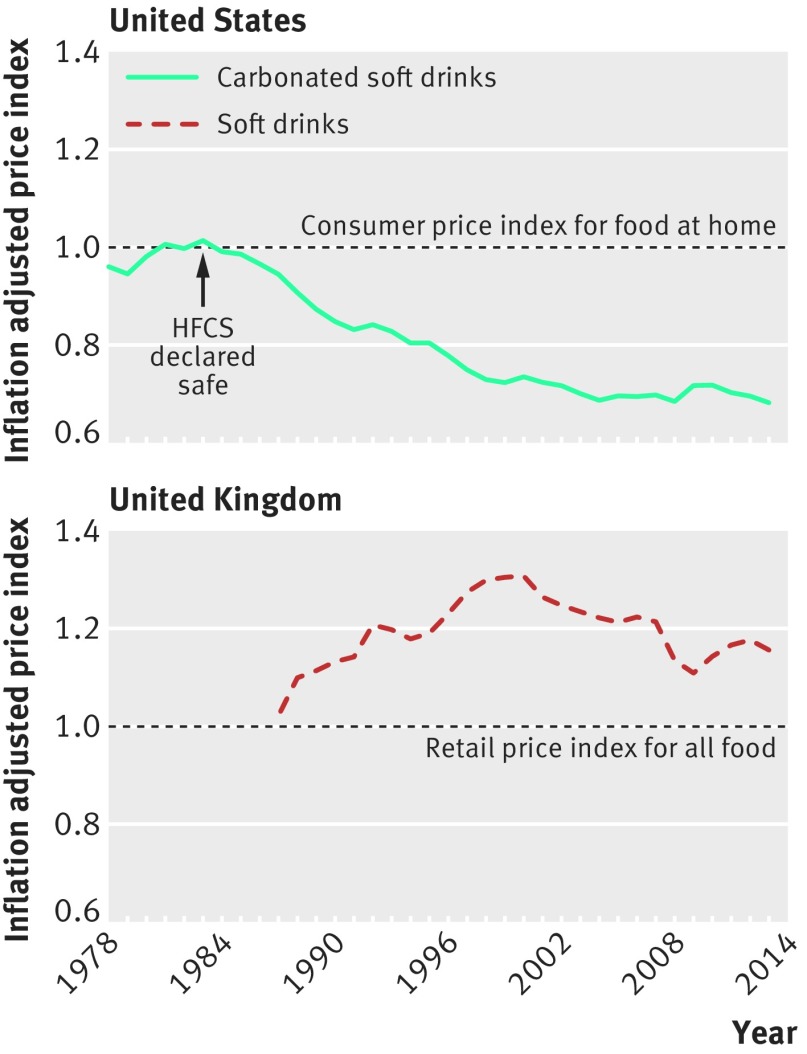
**Fig 2 (top)** US consumer price index for carbonated beverages adjusted for inflation based on the index for all food and drink at home, 1978-2014; baseline year set to 1978. Arrow indicates the year high fructose corn syrup (HFCS) was declared generally recognised as safe. **(Bottom)** UK retail price index for soft drinks adjusted for inflation based on the index for all food and drink, 1987-2013; baseline year set to 1987 (data from Bureau of Labor Statistics and Office of National Statistics)

Substantially cheaper sucrose and high fructose corn syrup may also lead to greater marketing of foods high in sugars because these foods will remain very profitable—and potentially more profitable than in the past. This may encourage industry to resist regulations designed to reduce the use of sugars.

The effects of the reforms are likely to be felt beyond Europe. It is intended that the sugar reforms will open up the world market, particularly in developing countries, for European processed food, which will become cheaper to produce as sugar prices fall. The EU Trade Commission and the UK Department for Environment, Food and Rural Affairs (Defra) have supported these reforms because of the opportunities they bring for the European and UK processed food industry.^20^ ^27^ Defra has stated that “the boom in global demand for western-style foods is creating huge opportunities for growth in [the sucrose and food manufacturing] sector which [the UK] should not hold back.”^27^

## Good health needs good agriculture policy

European agricultural policy, and particularly sugar liberalisation, has largely not considered health.^13^ ^14^ Although some weak public health objectives have been incorporated in recent years, health is not listed as one of the policy’s five main objectives.^28^ ^29^ ^30^ The structuring and sequencing of the reforms in 2006 and 2013 indicates that they were designed to benefit industry rather than public health.^20^ There has been no pause to consider the broader health implications of sugar reform, even though from the outset the European Commission forecasted that sugar consumption would increase as a result.

Tension between agricultural and nutritional policies is widespread, not only in Europe. In most countries agricultural and health ministries are separate with little interaction. For example, US agricultural policy has heavily encouraged overproduction of corn since the 1970s and in doing so has contributed to large scale production and consumption of high fructose corn syrup, conflicting with the health goals of reducing obesity and type 2 diabetes.^1^ ^16^

However, consensus is growing that agricultural policy is integral to population health.^12^ ^13^ ^14^ ^15^ ^16^ ^17^ ^18^ ^19^ Examples of good practice exist. The North Karelia project in Finland instituted changes in agriculture, including a switch from dairy to fruit production and introducing rapeseed oil production. These changes, alongside other initiatives, were associated with improvements in population diet and reduced cardiovascular disease.^18^ Poland removed dairy and other animal fat subsidies in the 1990s, which is credited with contributing to an observed fall in coronary heart disease.^17^

The timing of the sugar reform is particularly unfortunate, creating a tension between agricultural policy and health policy and generating mixed signals for the food industry. There is a risk that ongoing and proposed measures designed to reduce sugar consumption (such as reformulation to remove sugar, taxes on sugar sweetened drinks, and marketing restrictions) could be undermined by larger trends in price and production of sugars in Europe.

## Possible effects on health inequalities

There is already a socioeconomic gradient in sugar consumption among adults and a similar gradient in consumption of sugar sweetened drinks, which are a major source of added sugars (fig 3). Foods containing high levels of sugar are among the cheapest foods.^31^ Any reformulation to increase sugars in processed foods is unlikely to happen equally across all product lines. Cheaper processed food items, marketed on price rather than quality, may be most liable to reformulation to incorporate more sugars. These cheaper foods are purchased and consumed more often by people in lower socioeconomic groups,^32^ who are more price sensitive consumers.^33^ Consequently, this reform may disproportionately increase sugar consumption among lower socioeconomic groups, contributing to widening health inequalities.

**Figure fig3:**
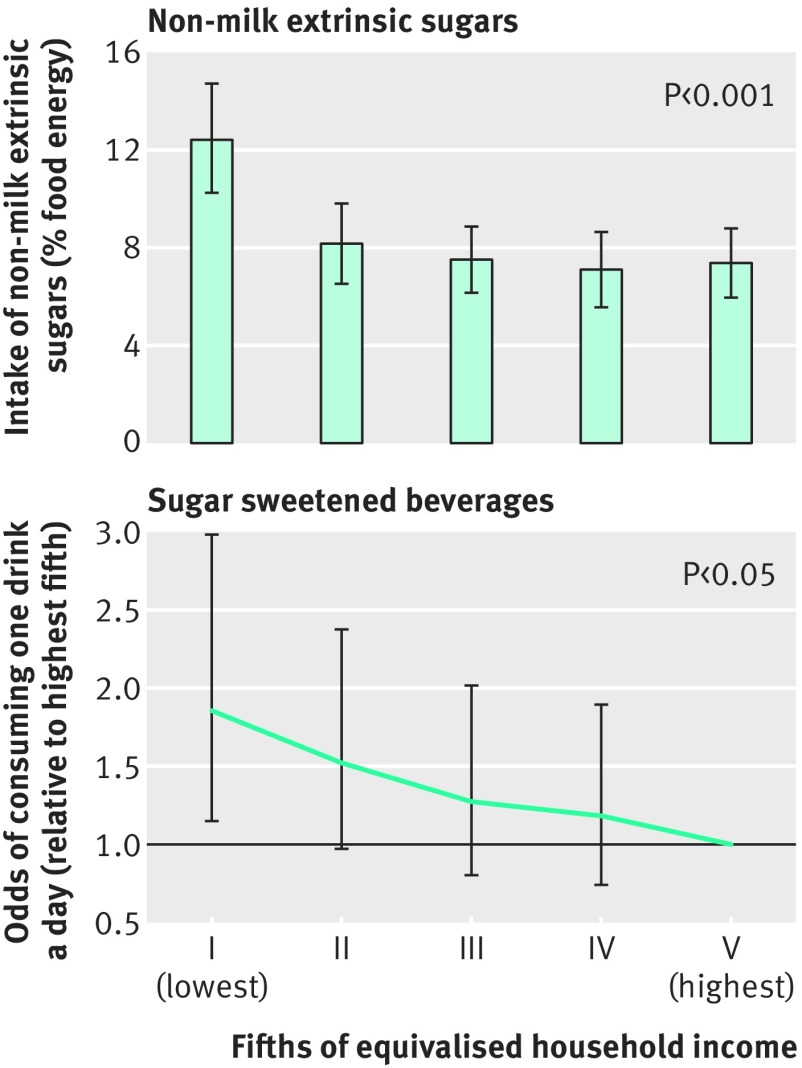
**Fig 3** Estimated mean intake (95% confidence intervals) of non-milk extrinsic sugars by equivalised fifths of household income for adults aged 19-64 (top) and odds of consuming a 165 mL portion of sugar sweetened beverage a day (bottom) for adults aged 19-64 in National and Diet Nutrition Survey.^7^ Estimated intake and odds are survey weighted and adjusted for age, sex, race, survey year, and total dietary energy intake. P values are for differences between the lowest and highest fifths

## Messages for policy makers

Since agriculture polices can shape food consumption and nutrition,^17^ ^18^ ^19^ they should explicitly integrate health. We should aspire to agricultural policies that promote a healthier diet, which can also deliver improvements in sustainability.^34^ Agricultural policies should be subject to full and meaningful health impact assessments to estimate the scale of potential population health effects and help identify solutions to mitigate health harms. No such assessment of the sugar reforms was undertaken. Although challenging, the relative success of health impact assessments in transport and integrating health into transport decision making suggests it is achievable.^35^ ^36^ ^37^

Given financial pressures on industry to reformulate foods to incorporate more sugar (or at least maintain existing formulation), it may be necessary for governments to mandate targets for reducing sugar contents of processed foods and implement robust systems for monitoring compliance. It will also be important to monitor food prices, diet, and health to determine the effects the reforms have on the cost and availability of foods, sugars in the food supply, and diets, including patterning of consumption among socioeconomic groups.

## Conclusions

Greater attention must be paid to the role agricultural policy plays in determining the price, availability, and consumption of sugars, especially as recent policy changes could increase consumption, particularly among the lowest socioeconomic groups. Europe and the UK must explore a set of short to medium term responses to address the projected increase of sugars in the food supply. In the longer term, they must integrate agriculture and health policies to help begin to address the larger structural factors affecting diet and population health.

**Key messages**

Reforms to the common agriculture policy will lower the commodity price of sugar and liberalise production of high fructose corn syrup in 2017
These changes have the potential to increase sugar consumption, particularly among the lowest socioeconomic groups
Europe must explore short to medium term responses to the projected increase of sugars in the food supply such as mandatory reformulation targets and improved monitoring of food content, diet, and health
In the longer term we should ensure that agricultural policies promote a healthier diet

## Notes

Contributors and sources: EKA is researching the diet, nutrition, and obesity implications of agricultural and trade law. She contributed the legal analysis of agriculture and trade policies on sugar. OTM and PM both carry out research into diet and health. OTM contributed to the writing of the manuscript and policy analysis. PM contributed to the writing and quantitative analysis and supervised the project. EKA is guarantor.

Provenance and peer review: Not commissioned; externally peer reviewed.

## Notes

Cite this as: *BMJ* 2015;351:h5085

## Related links

### thebmj.com

- See our interactive infographic on sugar’s web of influence at bmj.co/sugar
